# Induced metamorphosis in crustacean y-larvae: Towards a solution to a 100-year-old riddle

**DOI:** 10.1186/1741-7007-6-21

**Published:** 2008-05-20

**Authors:** Henrik Glenner, Jens T Høeg, Mark J Grygier, Yoshihisa Fujita

**Affiliations:** 1 Department of Biology, University of Copenhagen, Universitetsparken, DK-2100 Copenhagen, Denmark; 2 Lake Biwa Museum, Oroshimo 1091, Kusatsu, Shiga 525-0001, Japan; 3 Department of Chemistry, Biology and Marine Science, University of the Ryukyus, Nishihara, Okinawa 903-0213, Japan

## Abstract

**Background:**

The y-larva, a crustacean larval type first identified more than 100 years ago, has been found in marine plankton samples collected in the arctic, temperate and tropical regions of all oceans. The great species diversity found among y-larvae (we have identified more than 40 species at our study site alone) indicates that the adult organism may play a significant ecological role. However, despite intense efforts, the adult y-organism has never been identified, and nothing is therefore known about its biology.

**Results:**

We have successfully and repeatedly induced metamorphosis of y-larvae into a novel, highly reduced juvenile stage by applying the crustacean molting hormone 20-HE. The new stage is slug-like, unsegmented and lacks both limbs and almost all other traits normally characterizing arthropods, but it is capable of vigorous peristaltic motions.

**Conclusion:**

From our observations on live and preserved material we conclude that adult Facetotecta are endoparasitic in still to be identified marine hosts and with a juvenile stage that represents a remarkable convergence to that seen in parasitic barnacles (Crustacea Cirripedia Rhizocephala). From the distribution and abundance of facetotectan y-larvae in the world's oceans we furthermore suggest that these parasites are widespread and could play an important role in the marine environment.

## Background

Crustacean y-larvae (subclass Facetotecta) were first described from marine plankton in the late 1800s [1,2], and they have since been recorded from the arctic to the tropical waters of all oceans [3,4]. The adult organisms have never been identified, and the Facetotecta is the only crustacean group with a formal taxonomy based solely on larval stages [5,6]. At our study site, Sesoko Island near Okinawa, Japan, the enigmatic y-larvae form a significant and diverse component in the plankton. Based on previous extensive sampling at Sesoko Island [7] and on an extensive collection of mounted final naupliar exuviae (obtained from individual rearing surveys from 1996 to 2004) we found that more than 40 morphological types of y-larvae occur there, often abundantly and all representing undescribed species. Ontogenetic, morphological and molecular evidence all point to a taxonomic relationship with the crustacean class Thecostraca. These comprise the well-known barnacles (Cirripedia) and the Ascothoracida, but the Facetotecta forms a separate subclass and is not an ingroup in any of these two taxa [5,8,9]. Thecostracan crustaceans have sessile adults and a development comprised of a series of nauplius larvae and a final stage, the cyprid, specialized for attachment [10]. Similarly, the developmental sequence hitherto known for y-larvae (Facetotecta) comprises a series of (five) naupliar instars and a succeeding stage, the y-cyprid (Additional file 1), which is obviously adapted for settlement [5,11]. Among the related groups, the Ascothoracida are parasites in anthozoans and echinoderms while the Cirripedia, mostly comprised of suspension feeding organisms, also include the Rhizocephala, one of the most specialized groups of parasites within Crustacea [12,13]. This has prompted speculations that the Facetotecta are also parasitic [10], whence a study of the metamorphosis of their larvae could yield crucial information about the unknown adults. However, until now it has not been possible to rear y-larvae past the cyprid stage [3].

## Results

**Figure 1:**
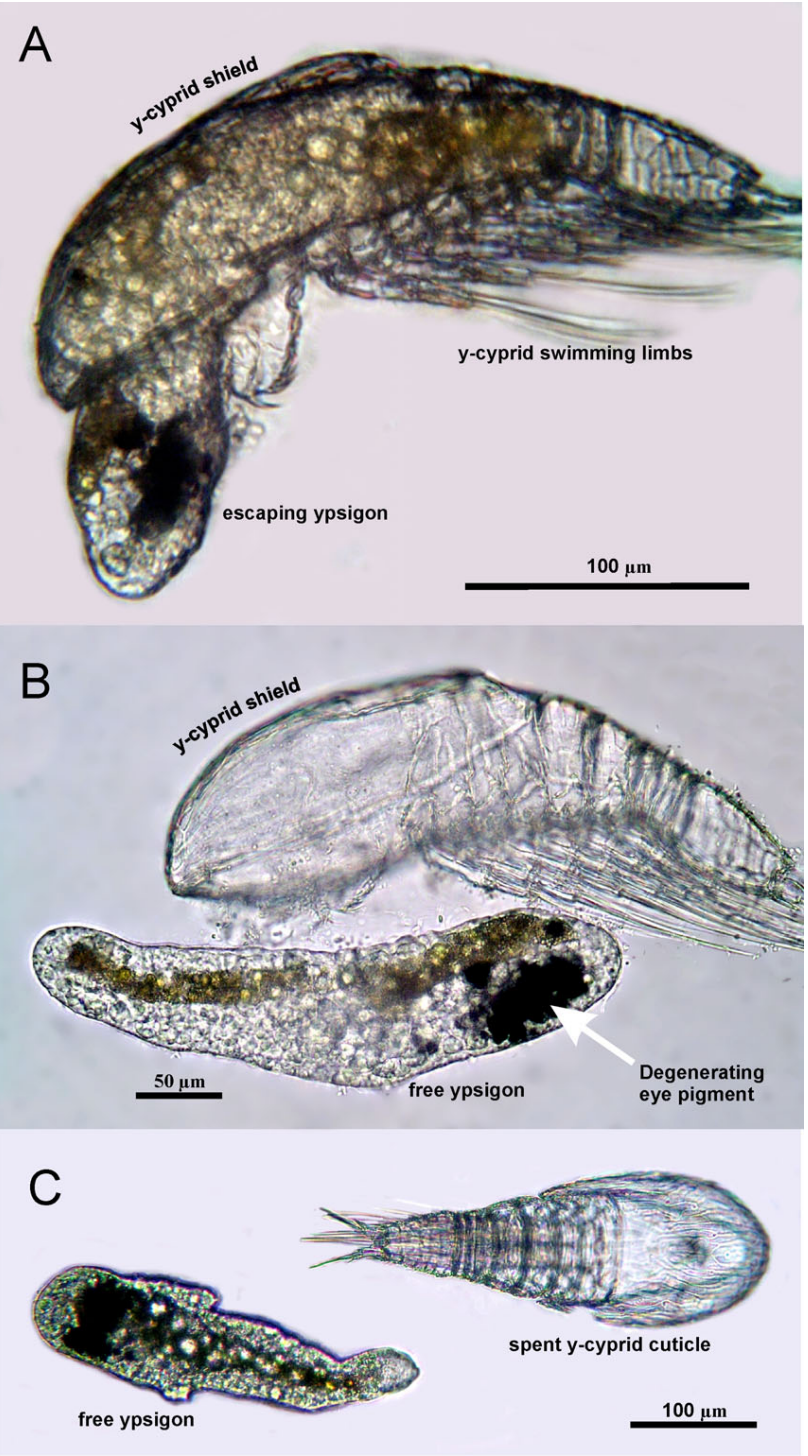
**Metamorphosis in y-larvae (Crustacea Facetotecta)**. (A) Light microscopy. Lateral view of a juvenile facetotectan (ypsigon stage) after 28 hours exposure to 20-hydroxyecdysone (20-HE), leaving the molted cuticle of the preceding y-cypris stage. The pigment of the degenerating compound eyes is seen anteriorly in the ypsigon. The shield and swimming limbs of the cyprid are left behind. (B) Lateral view of a free ypsigon after escape from its y-cypris 26 hours after exposure to 20-HE. Note the absence of segmentation and limbs. The elongated brownish body may represent the vestigial gut. (C) Dorsal view of free ypsigon disconnected from the empty y-cypris cuticle.

**Figure 2:**
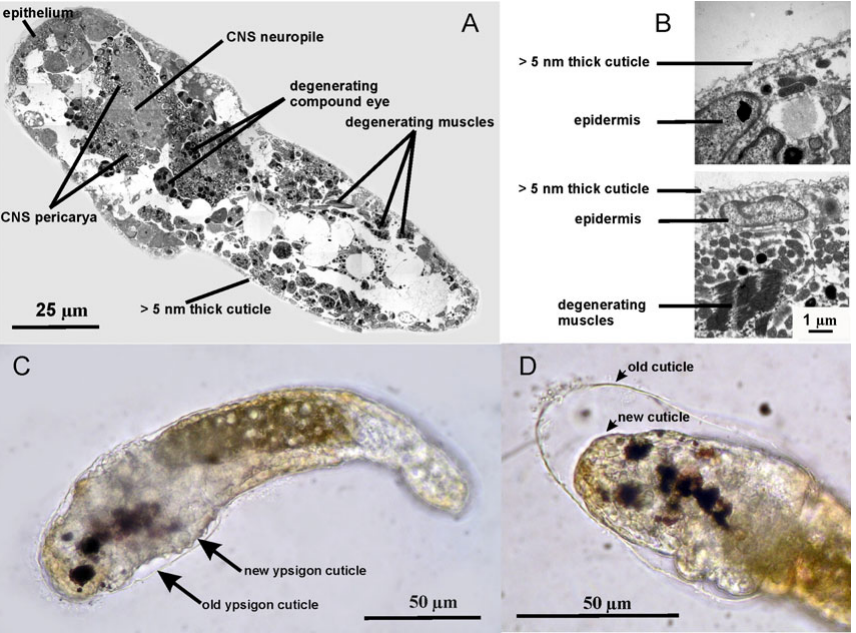
**Morphology of the ypsigon stage (Crustacea Facetotecta)**. (A) Horizontal transmission electron microscopy section through the ypsigon shown in Figure 1C. Collage of multiple frames. (B) Two high-magnification frames from A. The epidermis is clearly overlain by an ultrathin cuticle. (C) Light microscopy. Lateral view of a molting ypsigon, 48 hours after escape from the y-cyprid. (D) Light microscopy. Lateral view of the specimen in C, but 4 hours later. The first ypsigon cuticle, laid down while still within the y-cyprid, is now being shed and replaced by the new one. The interior structure of the ypsigon does not change noticeably during the molt.

**Figure 3:**
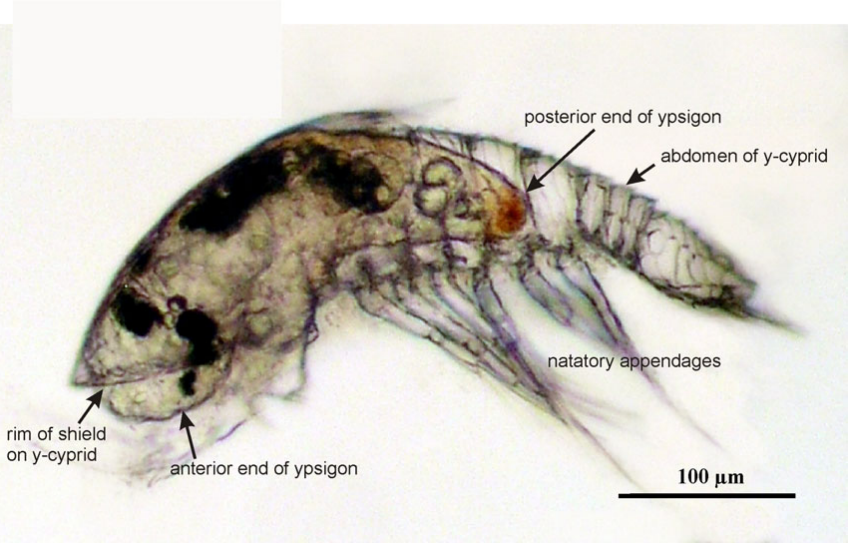
**Ypsigon within the y-cyprid**. A fully formed ypsigon located within the y-cyprid and just starting to escape through a hole near the anterior end. All y-cyprid tissues are included in the new ypsigon stage, leaving behind only the empty cuticle and all appendages of the y-cyprid. Note how the thorax (with natatory appendages) and abdomen of the y-cyprid shows distinct segmentation, while the slug-shaped ypsigon is entirely unsegmented.

**Figure 4:**
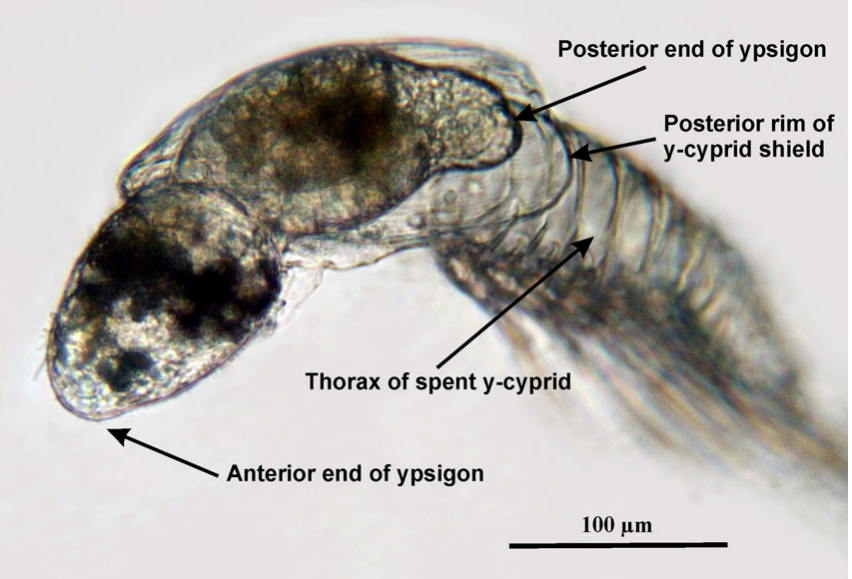
**'Plump' type ypsigon escaping from the y-cyprid**. Ypsigon escaping from the y-cyprid after 28 hours exposure to 20-hydroxyecdysone. This 'plump' type of ypsigon differs from the more elongated forms shown in Figures 1–3 and Additional files 1, 2, 3, 4, 5. It illustrates that the different 'species' of y-larvae recognized from molted naupliar skins (exuviae) is at least to some extent matched by variations in the shape of the juvenile ypsigon stage.

**Figure 5:**
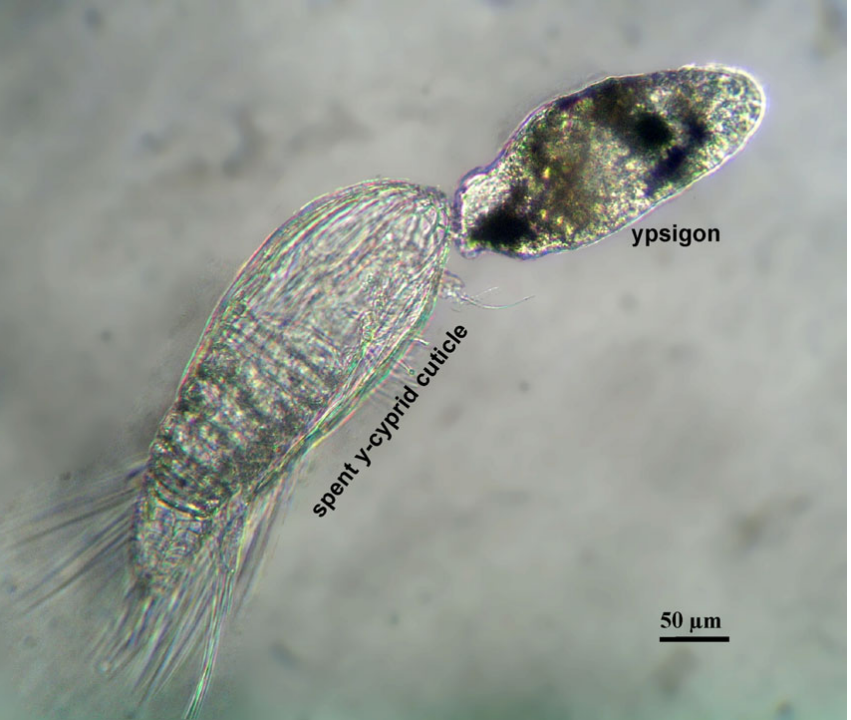
**'Plump' type ypsigon**. A 'plump' type of ypsigon (as in Figure 4), just after escape from its y-cyprid.

Exposure to 20-HE at concentrations within the effective range (1.04–2.08 μM) induced y-cyprids to metamorphose into a new and unexpected instar (Figure 1). This 300–400 μm long juvenile has a greatly simplified morphology and is surrounded by an extremely thin (<5 nm) cuticle. We documented this metamorphosis from several 'species' of y-cyprids by still photography, video and transmission electron microscopy (TEM); see Figures 1, 2, 3, 4, 5 and Additional files 1, 2, 3, 4, 5. Following exposure to the hormone, metamorphosis begins after 12–24 hours by a retraction of all, or nearly all, cyprid tissues into a compact body anteriorly, which is then surrounded by the newly secreted, thin cuticle. While still inside the y-cyprid, the body of the juvenile begins to exhibit contractions and bending motions. Aided by these motions it exits from the y-cyprid after 31–72 hours through a hole between the bases of the antennules in a process lasting 2–4 hours (Figure 1A and Additional file 2). The molted juvenile has been named the 'ypsigon', which refers to 'ypsilon' (the Greek letter y) and 'gonos' (Greek for 'larva'). The ypsigon is unsegmented, slug-like and lacks appendages (Figures 1B and 1C). The body motions persists after escape and allow the ypsigon to crawl on the bottom of the culture vessel and move several body lengths away from the spent y-cyprid within a few minutes (Additional files 3 and 4).

Like the preceding y-nauplius and y-cyprid stages, the ypsigon lacks a functional gut, but an elongated, dark mass of cells filled with fat globules and extending through most of the body may represent a vestige of this organ system (Figures 1B and 2C). Within the ypsigon body the muscles and compound eyes of the preceding y-cyprid stage are in a state of advanced degeneration (Figure 2A).

The ultrathin cuticle and the decomposing compound eyes are the only morphological indications of its arthropod affiliation (Figures 2A and 2B). According to the species tested, the resulting ypsigon could differ in morphology from being elongated to having a rather plump shape, but it always exhibited the same simplified structure (Figures 3, 4, 5). Ypsigons kept alive in culture for more than 24 hours passed through a molt that yielded another, second juvenile stage (Figures 2C and 2D). This second ypsigon stage had the same morphology as the first and continued to exhibit the same bending motions until they were preserved 48 hours after emerging from the y-cyprid (Additional file 5).

## Discussion

Within Arthropoda, the extreme morphological reduction seen in the new ypsigon stage is matched only by the early endoparasitic 'vermigon' stage recently discovered in certain parasitic barnacles, the Rhizocephala Kentrogonida [14,15]. Both the vermigon and the ypsigon are formed by comparable metamorphic molts, in which the epidermis withdraws from the old cuticle, closes around all organs in the body and thereby forms the new slug-shaped stage. In addition, both the vermigon and the ypsigon exhibit vigorous body movements after their escape from the cypris, and they have virtually the same reduced morphology, being surrounded only by an exceedingly thin and very pliable cuticle and lacking segmentation, appendages and sensory organs. Metamorphic stages of rhizocephalans, similar to those emerging from naturally settled larvae [13,16], have been produced when chemically inducing cyprids to metamorphose *in vitro *[17]. By analogy with the rhizocephalan vermigon [15,18] and our observation that the ypsigon does not change morphology at the first molt after escape, all evidence appears consistent with the ypsigon also being endoparasitic, eventually giving rise to a parasitic adult. This conclusion is also supported by comparison with the similarly apodous commiform larvae in the more distantly related entoniscid Isopoda [19,20]. However, while rhizocephalans are incontestable members of the barnacles (Cirripedia), facetotectans diverge as a separate subclass at the base of the thecostracan phylogenetic tree and must represent a wholly separate evolution of extremely specialized parasites [5,8,9]. The evolution of very similar, slug-shaped endoparasitic stages (vermigon, ypsigon) in two different lineages (Rhizocephala, Facetotecta) is therefore a stunning example of convergent evolution. Analogy with the Rhizocephala also leads to the prediction that the adult facetotectan will turn out to have a highly simplified structure and this may help to explain why they have escaped observation. Future efforts will focus both on culturing the ypsigon *in vitro *to more advanced stages and screening of the local fauna to identify the host animals.

## Conclusion

While we cannot yet identify the adult y-organism or its host, the new, highly aberrant and obviously endoparasitic stage in the life history of the Facetotecta takes us a major step towards the solution of an enigma that has puzzled zoologists for more than 100 years [2,4,10,21-23]. It has been documented that parasites can significantly affect the structure of ecosystems, especially when they are parasitic castrators and/or are affecting host behavior [24]. Our observation of more than 40 facetotectan 'species' at a single study site indicates that a hitherto unknown and diverse fauna of advanced endoparasites remain hidden and could play important roles in many marine habitats.

## Methods

### Field collection and handling of larvae

Plankton was caught several times daily in September and October of 2004 and 2005 from the pier of the Sesoko Laboratory, Tropical Biosphere Research Center, University of the Ryukyus, on the southeast coast of Sesoko Island (26°38'N 127°52'E). We used a hand-thrown net with a 62 μm mesh width, and complete daytime samples were sorted for larvae. Dusk and nighttime samples were prefiltered at 1000 μm to exclude larger organisms and make sorting easier. Samples were inspected and sorted under a dissection microscope with transmitted light. As in other thecostracan Crustacea, facetotectan species can have either feeding (planktotrophic) or non-feeding (lecithotrophic) nauplii, while cyprids never feed [16]. From each plankton catch, we isolated the non-feeding y-nauplii into a 30 mm diameter plastic Petri dish with coarsely filtered (62 μm) seawater; any y-cyprids were isolated separately. The dishes were maintained at ambient room temperature (24–28°C). Those containing nauplius batch cultures were inspected at least three times daily and dead larvae were removed. When noted, stage-five nauplii, identified by the development of the pigmented compound eyes of the y-cyprid, were isolated singly into Petri dishes. After each stage-five nauplius had molted into a y-cyprid, the shed naupliar cuticle (exuvium) was individually mounted on a glass slide in glycerine jelly. This allowed us to document, which morphological type or 'species' it represented based on our extensive, unpublished records and collections of mounted larvae from this area. Occasionally y-cyprids appeared in the batch cultures between examinations, but apart from these and a few caught directly from the plankton, the great majority of all y-cyprids used in our induced metamorphosis trials (around 400) can thus be backtracked to a known naupliar type and be allocated to species, when a formal taxonomy for the Facetotecta is eventually established [3,4,6,25].

### Experimental setup

For the induction of metamorphosis, single cyprids were introduced into standard plastic plates with 15 mm wells containing the test solutions and incubated at 24–28°C. A preliminary experiment was set up to test the effect of three different metamorphosis-inducing compounds on the y-cyprids. They were dissolved in Millipore-filtered seawater and used in concentrations tested previously on cyprids of parasitic barnacles (Rhizocephala) [17]: 3-isobutyl-1-methylxanthine (IBMX at 4.5 μM), 20-hydroxyecdysone (20-HE at 208 μM, 20.8 μM, 2.08 μM, 1.04 μM and 208 nM), and juvenile hormone (JH at 37.5 nM, 375 nM and 3.75 μM). This screening showed that only 20-HE was able to induce the y-cyprids to metamorphose, and another preliminary trial established that the most efficient concentration was between 1.04 and 2.08 μM. All subsequent experiments were conducted within this range.

### Photo/video documentation

All y-cyprids tested were used within 24 hours after the molt from the nauplius stage five. Well plates were inspected several times daily using an Olympus inverted microscope. Stages of metamorphosis were photographed with an Olympus C5050 digital camera and digital videos obtained with a Sony HAD Power Head. The plastic well plates do not allow optimum conditions for microphotography. To obtain better micrographs and videos, selected specimens were temporarily transferred to glass slides with seawater and observed using a Nikon Microphot with up to 40× objectives. Digital images were optimized for contrast and color using Corel X3 suite. The only other manipulation was to touch up the background, not the specimen, in some scenes to remove extraneous particles.

### Transmission electron microscopy

For TEM, selected stages of metamorphosing y-cyprids and free juveniles were fixed and stored in 2.5% glutaraldehyde in Millipore-filtered seawater buffered to pH 7.4 with 0.1 M sodium cacodylate. Following three rinses in the same buffer, specimens were postfixed in 1% OsO4, dehydrated with dimethoxypropane and embedded in TAAB 812 ('Epon') resin. Sections were mounted on slot grids, stained with uranyl-acetate and lead-citrate and inspected using a JEOL JEM-1011 transmission electron microscope fitted with a GATAN digital camera.

## Authors' contributions

JTH conceived the project, which was jointly planned in detail by JTH and MJG. MJG and YF were responsible for project logistics. Initial investigations were carried out jointly by JTH, MJG and YF. HG designed and conducted the experiments using 20-HE. JTH managed still and video photography and TEM techniques and assisted with the 20-HE experiments. YF managed larval culture and MJG the mounting of larvae and identification of 'species'. All authors participated in the analysis of the results. The manuscript was drafted by HG and JTH and all authors contributed jointly to the completed version.

## Supplementary Material

Additional file 1 **Video 1 – Swimming y-cyprid**. A y-cyprid swimming using its six pairs of thoracic appendages. The clip was recorded from a y-cyprid mounted under a cover slip, whence the body motions do not fully reflect natural swimming. When free in the water column, the y-cyprid swims almost continuously along a more or less straight line. Click here for file

Additional file 2 **Video 2 – Metamorphosing y-cyprid**. Three phases in the metamorphosis of a single y-larva individual. The first phase shows the ypsigon about to escape, but with the posterior part of its body still located within the shell of the spent y-cyprid. The next phase shows the actual exit of the ypsigon from the y-cyprid. The third phase shows the free ypsigon shortly afterwards, when it has moved some distance away from the spent y-cyprid. Click here for file

Additional file 3 **Video 3 – Free ypsigon**. An ypsigon that has just escaped from its y-cyprid. It exhibits moderate body movements (same specimen as shown in Figure 1B). Click here for file

Additional file 4 **Video 4 – Free ypsigon**. A free ypsigon that has moved away from its y-cyprid and now exhibiting very vigorous body movements. Click here for file

Additional file 5 **Video 5 – Molting ypsigon**. Ypsigon, 48 hours after escape from the y-cyprid. Now in the process of molting, while still exhibiting vigorous movements of the body (this is the same specimen as shown in Figures 2C and 2D). Note how the first ypsigon cuticle is clearly separated from the new one by a wide empty space. The internal structure of the ypsigon has not changed noticeably during the molt. Click here for file

## References

[B1] Hensen V (1887). Über die Bestimmung des Planktons oder des im Meere treibenden Materials an Pflanzen und Thieren. Bericht der Kommision zur wissenschaftlichen Untersuchung der deutschen Meere.

[B2] Hansen HJ (1899). Die Cladoceren und Cirripedian der Plankton-expedition. Ergeb Plankton-Expedit Humboldt-Stift.

[B3] Grygier MJ, Forest J (1996). Classe des Thécostracés (Thecostraca Gruvel, 1905). Sous-Classe des Facetotecta (Facetotecta Grygier, 1985). Traité de Zoologie Part 2 Crustacés, Généralités (suite) et Systématique (1re partie).

[B4] Høeg JT, Kolbasov GA (2003). Facetotectan larvae from the White Sea with the description of a new species (Crustacea: Thecostraca). Sarsia.

[B5] Høeg JT, Kolbasov GA (2002). Lattice organs in y-cyprids of the Facetotecta and their significance in the phylogeny of the Crustacea Thecostraca. Acta Zool.

[B6] Belmonte G (2005). Y-Nauplii (Crustacea, Thecostraca, Facetotecta) from coastal waters of the Salento Peninsula (south eastern Italy, Mediterranean Sea) with descriptions of four new species. Mar Biol Res.

[B7] Grygier MJ (1991). Facetotecta ('Y-larvae'): One day's catch in Okinawa, Japan (Crustacea: Facetotecta). Mem Queensl Mus.

[B8] Grygier MJ (1987). New records, external and internal anatomy, and systematic position of Hansen's Y-larvae (Crustacea, Maxillopoda, Facetotecta). Sarsia.

[B9] Perez-Losada M, Høeg JT, Kolbasov GA, Crandall KA (2002). Reanalysis of the relationships among the Cirripedia and the Ascothoracida and the phylogenetic position of the Facetotecta (Maxillopoda: Thecostraca) using 18S rDNA sequences. J Crustacean Biol.

[B10] Brusca CB, Brusca GJ (2003). Invertebrates.

[B11] Itô T (1990). Naupliar development of *Hansenocaris furcifera *Itô (Crustacea: Maxillopoda: Facetotecta) from Tanabe Bay, Japan. Publ Seto Mar Biol Lab.

[B12] Grygier MJ, Høeg JT, Rohde K (2005). Ascothoracida (Ascothoracids). Marine Parasites.

[B13] Høeg JT, Glenner H, Shields JJ, Rohde K (2005). Cirripedia Thoracica and Rhizocephala (barnacles). Marine Parasites.

[B14] Glenner H, Høeg JT (1995). A new motile, multicellular stage involved in host invasion by parasitic barnacles (Rhizocephala). Nature.

[B15] Glenner H, Høeg JT, O'Brien JJ, Sherman TD (2000). The invasive vermigon stage in the parasitic barnacles *Loxothylacus texanus *and *L. panopaei *(Sacculinidae): closing of the rhizocephalan life cycle. Mar Biol.

[B16] Høeg JT, Møller OS (2006). When similar beginnings lead to different ends: constraints and diversity in cirripede larval development. Invertebr Reprod Dev.

[B17] Glenner H, Brodin B (1997). Phorbol ester-induced metamorphosis in the parasitic barnacle, *Loxothylacus panopaei*. J Mar Biolog Assoc U K.

[B18] Glenner H (2001). Cypris metamorphosis, injection and earliest internal development of the Rhizocephalan *Loxothylacus panopaei *(Gissler). Crustacea: Cirripedia: Rhizocephala: Sacculinidae. J Morphol.

[B19] Veillet A (1945). Recherches sur le parasitisme des crabes et des galathées par les rhizocéphales et les épicarides. Ann Inst Oceanogr Monaco.

[B20] Kuris AM, Poinar GO, Hess T (1980). Post-larval mortality of the endoparasitic isopod castrator *Portunion conformis *(Epicaridea: Entoniscidae) in the shore crab, *Hemigrapsus oregonensis*, with a description of host response. Parasitology.

[B21] Bresciani J (1965). Nauplius "y" Hansen. Its distribution and relationship with a new cypris larva. Vidensk Meddr dansk naturh Foren.

[B22] Schram FR (1986). Crustacea.

[B23] Conway DVP, White RG, Hugues-Dit-Ciles J, Gallienne CP, Robins DB (2003). Guide to the coastal and surface zooplankton of the south-western Indian Ocean. Occasional Publ Mar Biolog Assoc U K.

[B24] Kuris AM, Lafferty KD (1994). Community structure – larval trematodes in snail hosts. Annual Review of Ecology and Systematics.

[B25] Kolbasov GA, Grygier MJ, Ivanenko VN, Vagelli AA (2007). A new species of the y-larva genus *Hansenocaris *Itô, 1985 (Crustacea: Thecostraca: Facetotecta) from Indonesia, with a review of y-cyprids and a key to all their described species. Raffles Bull Zool.

